# Mapping the Epidemiology of Yaws in the Solomon Islands: A Cluster Randomized Survey

**DOI:** 10.4269/ajtmh.14-0438

**Published:** 2015-01-07

**Authors:** Michael Marks, Ventis Vahi, Oliver Sokana, Elliot Puiahi, Alex Pavluck, Zaixing Zhang, Tenneth Dalipanda, Christian Bottomley, David C. Mabey, Anthony W. Solomon

**Affiliations:** Clinical Research Department, Faculty of Infectious and Tropical Diseases, London School of Hygiene and Tropical Medicine, London, United Kingdom; The Hospital for Tropical Diseases, Mortimer Market Centre, Mortimer Market, London, United Kingdom; Ministry of Health and Medical Services, Honiara, Solomon Islands; The Task Force for Global Health, Atlanta, Georgia; World Health Organization, Western Pacific Region Office, Honiara, Solomon Islands; Department of Infectious Diseases Epidemiology, London School of Hygiene and Tropical Medicine, London, United Kingdom

## Abstract

Yaws, a non-venereal treponemal disease, is targeted for eradication by 2020 but accurate epidemiological data to guide control programs remain sparse. The Solomon Islands reports the second highest number of cases of yaws worldwide. We conducted a cluster randomized survey of yaws in two provinces of the Solomon Islands. One thousand four hundred and ninety-seven (1,497) children 5–14 years of age were examined. Clinical signs of active yaws were found in 79 children (5.5%), whereas 140 children (9.4%) had evidence of healed yaws lesions. Four hundred and seventy (470) (31.4%) children had a positive *Treponema pallidum* particle agglutination assay (TPPA). Two hundred and eighty-five (285) children (19%) had a positive TPPA and rapid plasma regain assay. Risk of yaws increased with age and was more common in males. The prevalence of yaws at village level was the major risk factor for infection. Our findings suggest the village, not the household, should be the unit of treatment in the World Health Organization (WHO) yaws eradication strategy.

## Introduction

Yaws is a non-venereal, endemic treponemal infection caused by *Treponema pallidum pertenue*^1^ and is closely related to *T.p. pallidum*, the causative agent of venereal syphilis.^2^ Unlike syphilis, yaws is generally a disease of children^3^ living in poor, rural settings and is found mostly in warm and humid environments.^4^ Infection predominantly results in lesions of the skin and bone.^5^ Tertiary yaws causes disfiguring lesions of the face but neurological and cardiovascular manifestations (which can appear in advanced syphilis) are not thought to occur. A major campaign in the 1950s was responsible for a significant reduction in the prevalence of disease worldwide, but following the dismantling of vertical control programs, the number of cases subsequently rebounded, and yaws now represents a significant public health problem in Africa, South-East Asia, and the Pacific.^6^–^10^

In a single-center study in Papua New Guinea, azithromycin was shown to be equivalent to benzathine-penicillin in the treatment of both primary and secondary yaws.^11^ The discovery of an orally effective agent prompted the World Health Organization (WHO) to plan a renewed effort to eliminate yaws using community mass treatment.^12^

Despite optimism, clinical and epidemiological data on yaws remain sparse. Many countries report only clinical cases without laboratory confirmation and there are inadequate data to guide control programs in many countries. Previous studies have shown that the majority of clinical cases occur in children < 15 years of age, and have also suggested a preponderance of cases in males.^3^,^8^,^13^ It is also recognized that for every clinical case there are 5–6 individuals with serological evidence of infection but no clinical manifestations.^8^,^13^

The Solomon Islands, a Pacific archipelago of ∼100 inhabited islands, is endemic for yaws, and in 2010 the country reported 20,635 cases of yaws, the second highest number of cases in the world after Papua New Guinea, which lies immediately to the north.^5^ We conducted a cluster randomized community survey in two provinces of the Solomon Islands to establish the prevalence of yaws infection and disease, and to explore risk factors associated with infection and disease at community level.

## Materials and Methods

### Participant recruitment.

The study was undertaken in Western and Choiseul Provinces of the Solomon Islands in September and October 2013, alongside programmatic trachoma mapping activities.^14^ The two provinces were considered as two separate evaluation units. We defined clusters either as villages or as groups of villages to obtain 50 clusters of approximately equal size. Twenty-five clusters were selected at random in each province. In each cluster, a complete census of all households was obtained and 30 households were selected using simple random sampling. In selected households children 5–14 years of age were invited to participate. Where selected households were unoccupied, an alternative household was randomly selected. Written informed consent was obtained from the head of each household by staff fluent in the local dialect. Assent was obtained from all children. We calculated that a sample size of 1,022 would allow us to detect a prevalence of infection of 10% with absolute precision of 3% assuming a design effect of 2.5.

### Data collection.

Household-level data were collected on location (recorded using the smartphone global positioning system) and Water, Sanitation and Hygiene (WASH) variables, including source of water and the presence or absence of hand washing facilities and latrines.

Participant-level data were collected on demographics and clinical features of yaws. The survey team completed a standardized history and examination, collecting information on bone and joint symptoms, yaws treatment history, and the number, location, and duration of skin lesions. Skin lesions were classified using the WHO pictorial yaws classification scheme. All data were collected using the LINKS software package on Android smartphones.^15^

The survey team performed venipuncture on all individuals, regardless of clinical features, and obtained a serum sample for diagnostic testing. Samples were transferred to the National Referral Hospital in Honiara where they were frozen. Samples were transferred on dry ice for diagnostic testing at the London School of Hygiene & Tropical Medicine (LSHTM).

### Laboratory studies.

Sera were tested using both the *Treponema pallidum* particle agglutination assay (TPPA, Mast Diagnostics, Merseyside, UK) and a quantitative rapid plasma regain test (RPR, Deben Diagnostics, Ipswich, UK), at LSHTM, by an operator masked to clinical findings.

### Ethics.

Ethical approval for the study was granted by the ethics committees of the Ministry of Health and Medical Services in the Solomon Islands (HRC 13/10) and LSHTM (6358) in the UK.

### Diagnosis.

A positive TPPA was taken as evidence of previous or current infection. A positive TPPA combined with a quantitative RPR titer of ≥ 1/4 was taken to represent dual-positive serology, suggestive of active infection. Individuals with a single ulcerative or papillomatous lesion and dual-positive serology were considered to have primary yaws. Individuals with skin or bony lesions consistent with secondary yaws and dual-positive serology were considered to have secondary yaws. Individuals with late manifestations (gondou, gangosa, or gummatous lesions) would have been considered to have tertiary yaws. Individuals with dual-positive serology but no current clinical signs were considered to have latent yaws.

### Analyses.

Logistic regression was used to estimate unadjusted and adjusted odds ratios (ORs) for factors associated with both TPPA positivity or dual seropositivity. We classified household size as less than or greater than the national average (5 residents) according to the 2009 census. There are no established criteria for classifying yaws endemicity based on serological findings. We adjusted previous treatment criteria, based on the number of clinical cases,^3^ by a factor of 5 to accommodate individuals with latent infection. For the purpose of analysis villages were classified as hypo-endemic (0–25% of surveyed individuals dual seropositive), meso-endemic (26–50% of individuals dual seropositive), or hyper-endemic (> 50% of individuals dual seropositive). Robust standard errors were used to calculate all confidence intervals (CIs) and *P* values to account for village-level clustering. All analyses were performed using Stata 13.1 (StataCorp, College Station, TX).

## Results

### Clinical findings.

**Figure 1.**
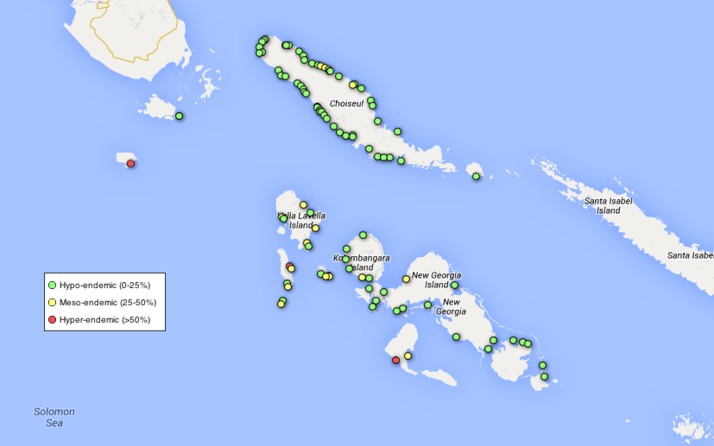
Map showing locations of surveyed villages. Villages where the seroprevalence of yaws was 0–25% (hypo-endemic), 25–50% (meso-endemic), and > 50% (hyper-endemic) are shaded green, yellow, and red, respectively. The predominance of coastal villages reflects the distribution of communities within the Solomon Islands.

One thousand four hundred and ninety-four (1,494) households in 981 villages were visited; there was no-one home, or no residents 5–14 years of age, in 656 households. One thousand four hundred and ninety-seven (1,497) children were enrolled from 98 villages and 838 households (Figure 1

). Demographic data on these individuals are shown in Table 1. More children were enrolled in Western province (*N* = 889) than Choiseul province (*N* = 608). Skin lesions, of any type, were found in 290 children (19.4%, 95% CI 14.3–25.6%).

In total, clinical signs suggestive of active yaws were found in 79 (5.5%, 95% CI 4.0–7.5%) children. Of these, 50 children (3.3%, 95% CI 2.4–4.6%) had skin lesions consistent with primary yaws, whereas 29 (2.0%, 95% CI 1.1–3.7%) had signs consistent with secondary yaws. Skin lesions consistent with secondary yaws were found in 19 (1.3%, 0.6–2.9%), bone lesions consistent with secondary yaws were found in 10 (0.7%, 95% CI 0.3–1.5%) children. One hundred and forty (140) children (9.4%, 95% CI 6.1–14.1%) had evidence of healed yaws lesions, of whom 7 also had evidence of active yaws. Two hundred and eighteen (218) children (15.2%, 95% CI 11.0–20.5%) reported treatment of yaws in the last 12 months.

### Serological findings.

**Figure 2.**
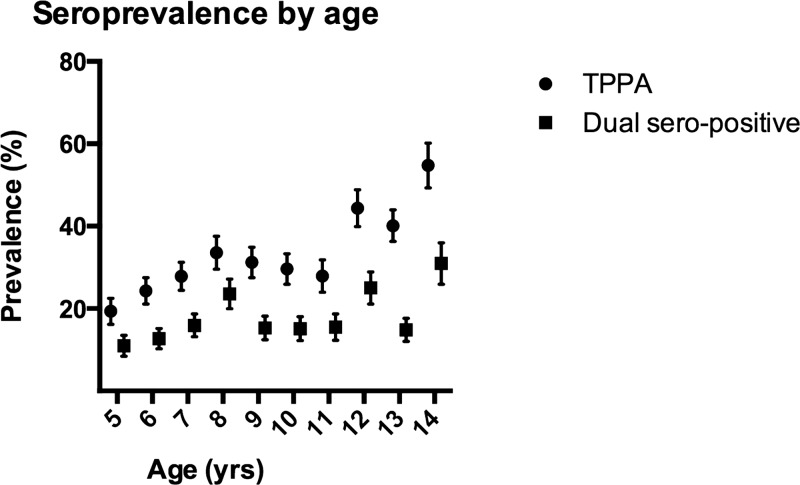
Seroprevalence by age prevalence (and 95% confidence interval [CI]) of *Treponema pallidum* particle agglutination (TPPA) assay and dual seropositivity (TPPA and rapid plasma regain [RPR] titer ≥ 1/4) by age group.

Four hundred and seventy children (470) had a positive TPPA, giving a seroprevalence of previous or current treponemal infection of 31.4% (95% CI 23.6–41.5%). As expected, the prevalence of TPPA positivity increased with age (Figure 2). In Western province, seroprevalence was 37.6% (95% CI 28.1–51.1%), as compared with 21.7% (95% CI 10.4–40.2%) in Choiseul province; after adjustment for clustering this difference was not statistically significant (*P* < 0.09).

Of individuals with a positive TPPA, 285 (60.6%) had an RPR ≥ 1/4, suggesting active treponemal infection. Dual seropositivity was therefore 19.0% overall, but varied significantly between Western Province (*N* = 186, 21.7%, 95% CI 14.6–30.9%) and Choiseul Province (*N* = 63, 10.4%, 95% CI 4.1–24.2%) (*P* = 0.11).

Twenty-four (30.4%) of 79 children with signs of active yaws and 40 (28.6%) of 140 children with signs of healed yaws were dually seropositive, compared with 14.8% of children without signs of active or healed yaws (*P* < 0.01 for both comparisons) (Table 2).

Based on serology data we classified 77 (78.6%) villages as non-endemic, 15 (15.3%) villages as endemic, and 6 (6.1%) villages as highly endemic. Eighty percent of dual-serologically positive individuals lived in a meso-endemic or hyper-endemic village.

### Risk factors.

In both unadjusted and adjusted analyses, age, sex, number of dual seropositive household contacts, degree of village endemicity, and access to hand washing facilities were all associated with both TPPA positivity and dual seropositivity, and active disease. Household size was not a risk factor for either infection or active disease (Tables 3 and 4).

## Discussion

This study confirms that yaws is endemic in the north-west of the Solomon Islands, with one-third of children in this area showing evidence of infection by TPPA. In keeping with national case reporting, we observed a difference in the prevalence of clinical signs of yaws and of both TPPA positivity and dual seropositivity between Western and Choiseul provinces, although this result was not statistically significant after accounting for clustering. As anticipated, there was significant clustering of both infection and disease. The degree of endemicity at village level was the strongest risk factor for both TPPA positivity and dual seropositivity, a finding that suggests communities, not households, should be the target of contact tracing and treatment as part of the WHO yaws eradication campaign in this population. Treatment failure with penicillin has previously been reported to be associated with the degree of endemicity at village level.^16^ Our data support the theory that reinfection, rather than true treatment failure, may partially explain this finding.

Seropositivity was strongly associated with the number of seropositive household contacts. It is possible that this association is a consequence of transmission to household contacts from an actively infected individual rather than a risk factor for initial infection per se. Longitudinal data would be needed to better explore this association.

As in previous studies, there were several individuals with serological evidence of infection for every individual with clinically apparent disease,^8^,^13^ and a large number of individuals with typical skin lesions who were not dually seropositive.^17^ Lesions not associated with positive serology may reflect early yaws before conversion, recent treatment of yaws with declining RPR titers, misclassification of the lesion by survey personnel, or alternative aetiologies of ulcerative skin lesions. *Haemophilus ducreyi* has recently been identified as a possible causative agent of yaws-like lesions in the Pacific region.^18^–^20^

As expected, the prevalence of TPPA positivity increased with age within the age group examined, and the study also confirmed previous reports^8^,^13^ that males are at slightly higher risk than females of both TPPA positivity and dual seropositivity. The reasons for this association are unclear. It is possible that the association with gender reflects an earlier onset of sexual intercourse in boys than girls, with misclassification of cases of syphilis as yaws in endemic communities. In our study, the association with gender remained when analyses were restricted to children < 10 years of age, making this kind of misclassification less likely.

An unexpected finding was the protective association between access to hand washing facilities and the risk of TPPA positivity and dual seropositivity. It is thought that yaws is spread by skin-to-skin contact when bacteria from a lesion enter by a breach in the skin.^3^,^21^ It is plausible that access to hand washing facilities promotes better overall skin health, reducing the number of small abrasions and lesions, which might serve as portals of entry for *T. pallidum* ssp. *pertenue*. It is also possible that this association is explained by residual confounding, from better general living conditions or access to health care facilities.

Although we did not examine adults during this survey, we did not see any cases of tertiary yaws in the communities we visited, a finding in keeping with a previous yaws survey in the Solomon Islands.^17^ The reasons why late stage manifestations are less common than previously reported are unclear, but improved access to health care and use of antibiotics with treponemocidal activity for other infections may partially account for this finding.

In summary, our data confirm that yaws is highly endemic in the Solomon Islands. Our study confirms previously suggested associations between gender and infection while also highlighting the need for further studies to examine the association with access to hand washing facilities. Prevalence of disease at a village level was shown to be the major risk factor for disease at an individual level, a finding that suggests the community should be the unit of treatment in response to the identification of cases of yaws, in the WHO yaws eradication strategy.^12^ Further studies are needed to investigate whether the associations shown in this study remain after community mass treatment with azithromycin.

## Figures and Tables

**Table 1:** Demographics

	Western Province	Choiseul Province
Number of children	889 (59.4%)	608 (40.6)
Number of households	494	344
Number of villages	45	53
Number clusters	25	25
Age (median, IQR)	9 (7–12)	9 (7–11)
Male	438 (49.3%)	323 (53.1%)
Household size (median, IQR)	5 (4–6)	5 (4–6)
Number positive TPPA individuals	334 (37.6%)	132 (21.9%)
Number dual seropositive individuals	186 (20.9%)	63 (10.4%)
Village classified as endemic	12 (26.7%)	3 (5.7%)
Village classified as highly endemic	4 (8.9%)	2 (3.8%)
Access to hand washing facilities	137 (15.4%)	52 (8.6%)

IQR = interquartile range; TPPA = *Treponema pallidum particle agglutination*.

**Table 2:** Serology results

		Overall	Western Province	Choiseul Province
Clinical evidence of primary yaws	n	50	38	12
	TPPA positive/RPR negative	1 (2%)	1 (2.6%)	0 (0%)
	TPPA positive/RPR < 1/4	3 (6%)	2 (5.3%)	1 (8.3%)
	TPPA positive/RPR ≥ 1/4	17 (34%)	11 (29.0%)	6 (50%)
Clinical evidence of secondary yaws	*N*	29	28	1
	TPPA positive/RPR negative	2 (6.9%)	2 (7.1%)	0 (0%)
	TPPA positive/RPR < 1/4	3 (10.3%)	3 (10.7%)	1 (0%)
	TPPA positive/RPR ≥ 1/4	7 (24%)	6 (21.4%)	1 (100%)
Clinical evidence of previous yaws	*N*	140	135	5
	TPPA positive/RPR negative	21 (15%)	21 (15.6%)	0 (0%)
	TPPA positive/RPR <1/4	25 (17.9%)	24 (17.8%)	1 (20%)
	TPPA positive/RPR ≥1/4	40 (28.6%)	40 (29.6%)	0 (0%)
No clinical evidence of active yaws	*N*	1278	688	590
	TPPA positive/RPR negative	59 (4.6%)	28 (4.1%)	31 (5.3%)
	TPPA positive/RPR < 1/4	122 (9.5%)	75 (10.8/%)	47 (8.0%)
	TPPA positive/RPR ≥ 1/4	188 (14.8%)	132 (19.2%)	56 (9.5%)

TPPA = *Treponema pallidum particle agglutination*; RPR = rapid plasma regain.

**Table 3:** Risk factors for TPPA positivity

		Illustrative prevalence data	Unadjusted OR	Adjusted OR
Age (years)*		5–9: 27.0% 10–14: 37.9%	1.14 (1.08–1.20) *P* < 0.001	
Sex		Male: 35.1% Female: 28.6%	1.35 (1.11–1.64) *P* = 0.002	
Household size		≤ 5: 29.8% > 5: 35.6%	1.30 (0.90–1.88) *P* < 0.160	
Number of household dual seropositive contacts†	0	22.9%		
	1	69.1%	7.52 (4.72–12.0) *P* < 0.001	3.92 (2.16–7.12) *P* < 0.001
	> 1	86.1%	20.80 (9.25–46.76) *P* < 0.001	10.74 (4.38–26.37) *P* < 0.001
Household dual seropositive contacts with skin ulcers‡		Absent: 30.7% Present 82.4%	10.54 (3.43–32.33) *P* < 0.001	7.52 (4.72–12.0) *P* < 0.001
Village§	Hypo-endemic	14.5%		
	Meso-endemic	67.0%	12.02 (6.99–20.68) *P* < 0.001	10.22 (6.35–16.42) *P* < 0.001
	Hyper-endemic	74.2%	17.01 (9.50–30.46) *P* < 0.001	20.51 (12.11–34.75) *P* < 0.001
Hand washing facilities¶		Absent 33.2% Present: 23.4%	0.62 (0.35–1.09) *P* = 0.095	0.62 (0.35–1.09) *P* = 0.095

* Relative increase in odds per year increase in age.

† TPPA positive with a reactive RPR titer ≥ 1/4. Adjusted for age, gender, household size, and village endemicity.

‡ TPPA positive with a reactive RPR titer ≥ 1/4 and an ulcerative skin lesion. adjusted for age, gender, household size, and village endemicity.

§ Adjusted for age, gender, household size, and household contacts.

¶ Hand washing facilities reported by family within 15 m of toilet/latrine. Adjusted for age, gender, and household size.

OR = odds ratio; TPPA = *Treponema pallidum particle agglutination*; RPR = rapid plasma regain.

**Table 4:** Risk factors for dual seropositivity

			Unadjusted OR	Adjusted OR
Age*		5–9: 15.4% 10–14:19.1%	1.08 (1.02–1.14) *P* = 0.006	1.08 (1.03–1.14) *P* = 0.003
Sex		Male: 19.5% Female: 14.4%	1.44 (1.38–1.83) *P* = 0.002	1.48 (1.17–1.88) *P* = 0.001
Household size		≤ 5: 16.0% > 5: 18.9%	1.22 (0.79–1.90) *P* = 0.362	
Number of household dual seropositive Contacts†	0	9.9%		
	1	46.4%	7.85 (4.12–14.97) *P* < 0.001	4.18 (2.12–8.25), *P* < 0.001
	> 1	59.3%	13.20 (6.40–27.21) *P* < 0.001	6.74 (2.72–16.70), *P* < 0.001
Household dual seropositive contacts with skin ulcers‡		Absent: 16.2% Present: 52.9%	5.82 (2.57–13.21) *P* < 0.001	7.52 (4.72–12.0) *P* < 0.001
Village§	Non-endemic	4.95%		
	Endemic	40.11%	12.85 (7.17–23.02) *P* < 0.0001	8.30 (5.36–12.86) *P* < 0.001
	Highly endemic	51.6%	20.46 (10.79–38.82) *P* < 0.0001	20.33 (11.88–34.80) *P* < 0.001
Hand washing facilities¶		Absent: 18.4% Present: 8.0%	0.39 (0.16–0.90) *P* = 0.029	0.39 (0.16–0.91) *P* = 0.029

* Increase in odds per year increase in age.

† TPPA positive with a reactive RPR titer ≥ 1/4. Adjusted for age, gender, household size, and village endemicity.

‡ Adjusted for age, gender, household size, and village endemicity.

§ TPPA positive with a reactive RPR titer ≥ 1/4 and an ulcerative skin lesion. adjusted for age, gender, household size, and village endemicity.

¶ Hand washing facilities reported by family within 15 m of toilet/latrine. Adjusted for age, gender, and household size.

TPPA = *Treponema pallidum particle agglutination*; RPR = rapid plasma regain.
